# Durum Wheat Couscous Grains: An Ethnic Mediterranean Food at the Interface of Traditional Domestic Preparation and Industrial Manufacturing

**DOI:** 10.3390/foods11070902

**Published:** 2022-03-22

**Authors:** Rifka Hammami, Reine Barbar, Marie Laurent, Bernard Cuq

**Affiliations:** 1 Field Crop Laboratory, National Institute of Agronomic Research of Tunisia, Carthage University, Ariana 2049, Tunisia; rifkahammami82@gmail.com; 2 L’Institut Agro Montpellier, UMR IATE, Université de Montpellier, 34060 Montpellier, France; reine.barbar@supagro.fr; 3 INRAe, UMR IATE, Université de Montpellier, 34060 Montpellier, France; marie.laurent@inrae.fr

**Keywords:** couscous, durum wheat, manufacturing processes, domestic preparation, history

## Abstract

Couscous is the product prepared from durum wheat semolina that agglomerates by adding water and undergoes physical and thermal treatment. Couscous is a traditional food from Mediterranean countries consumed for many centuries. Between ancestral domestic practices and industrial performance, the diversity of methods for couscous processing meets the needs of different consumers, whether they are concerned about preserving family culinary traditions or discovering innovative foods that respond to changing consumption patterns. In this work, we present the story of durum wheat couscous through several complementary visions and approaches: a “historical and societal“ approach to discover the origins of couscous, its migrations and its unifying role in Mediterranean societies; a “physicochemical” approach to describe the role of wheat components at the heart of couscous grains; a “technological” approach to compare domestic and industrial production of couscous; a “food science” approach to understand organoleptic characteristics of couscous grains; and a “consumer” approach to understand the motivations associated with the consumption of couscous.

The *Codex Alimentarius* [1] defined couscous as the product prepared from durum wheat semolina that agglomerates by adding water and undergoes physical and thermal treatment. Couscous is an inexpensive food staple with a long shelf life, and it can be simply prepared in different recipes: salads known as “taboulé” or traditional couscous dish. Knowledge, ancestral know-how and practices related to the production and consumption of couscous have been inscribed in 2020 on UNESCO’s list of Intangible Cultural Heritage [2]. Although considered as a traditional foodstuff, couscous still remains mysterious in terms of structuring mechanisms, elaboration processes and qualities of use. The present review addresses the manufacture of durum wheat couscous on traditional and industrial scales and the consumption of couscous in traditional and modern ways. Technical and scientific descriptions of couscous grains still remain patchy, with three book chapters [3,4,5]. The review was built on the basis of only about fifty publications and patents, a couple of students’ reports and PhD theses available to date and expertise from the authors’ laboratories.

## 1. The History of Couscous

### 1.1. Origins

Couscous is a very ancestral food product, nearly 2000 years old [3,5,6,7]. The combined origins between Berber food habits and those coming from Andalusia following the wave of mass migration of Muslims and Jews after Andalusia’s fall to the hands of Christians are among the many historical characteristics of couscous. Part of the origin of couscous is related to Numidians, the Berber population of Numidia. The culinary historian Lucie Bolens describes primitive pots that closely resemble the main cooking utensil of couscous, which is the couscoussier, found in Kabylia in tombs coming from the period of Berber king Massinissa, who ruled Algeria between 238 and 149 BC [7]. Archaeological evidence found in North Africa dating back to the 10th century covers the kitchen utensils needed to prepare couscous. Steaming grains over a broth in a special pot was first found in the west part of North Africa. The Berbers invented an original way to slightly moisten and roll the semolina of durum wheat into small spherical and succulent grains. These are light, fragrant and nourishing, giving a fluffy mass. Couscous was the basic cereal preparation of Berbers even before the Arabic conquest. Neither in the ancient world nor in the oriental Arab world are we aware of this way of treating grains. The first references issued about couscous were written in the 13th century in the North African cookbook.

### 1.2. Etymology

The worldwide known etymology of the word couscous may be derived from the Arabic word “*kaskasa*” meaning “to pound small” and also relating to the sound “*keskes*” arising from grains sieving, or also from the Berber “*seksu*”, meaning well rolled or well formed [6]. Couscous or “*seksu*” is pronounced “*koos-koos*” in the Berber language.

### 1.3. Migration

Couscous was spread by Arabs from the Mediterranean basin throughout Europe in the 17th century and moved to the Americas with Portuguese cargoes from Morocco. The Mediterranean basin sees extensive migratory flows: memories, senses, images, tastes and aromas travel with moving groups or individuals [5,8,9]. In the 16th century, couscous arrived in Turkey from Syria. In 1699, a letter mentioned couscous spreading in France and Brittany. Mediterranean migrants from Spain, Italy and Malta were part of the European population in the north of Africa. Since the end of the last century, the Maghreb couscous has become widespread in many countries of the world. Today, couscous is produced and eaten around the world.

### 1.4. French Context

Couscous had been present in France since the 19th century as the staple food of Kabyle people [9]. Couscous became a significant part of modern French cuisine on the way of the 19th-century colonial route. The first French people to settle in Maghreb were typically colonists of modest means who lived in rural areas [10]. During the 1970s, North African immigration intensified in order to support French economic growth. French law allowed for wives and children of immigrants to join them in France. This shift from the migration of male workers to the migration of families was a key factor in the enduring presence of couscous in France. The return of colonial populations to their native countries, following North African immigration, intensified Maghreb’s decolonization [5]. French nationals who colonized Algeria until the end of the Algerian War in 1962 adopted local produce until they could establish means of producing food [11]. During this period, the preparation of grains shifted from being a manual craft to an industrial operation, with the introduction of flour mills in Algeria (Maison Ricci in 1853 or Ferrero in 1970). Following independences between 1956 and 1962, the families of millers from North Africa typically settled in Marseille and developed the unexplored couscous manufacturing industry there [9]. In less than 50 years, couscous would become one of France’s three favorite savory dishes.

### 1.5. Durum Wheat Semolina

Couscous has not been developed at random and responded to a necessity. In North Africa, couscous is made from durum wheat semolina. Beyond agronomic and technological aspects, the durum wheat semolina carries a strong anchorage in the Mediterranean diet. Durum wheat semolina is the “traditional” raw material for the manufacture of couscous grains in Maghreb countries and the Mediterranean region because of the ideally suited color and cooking quality [12]. Sown in autumn to germinate in the rain, it is therefore called “winter wheat”. Durum wheat contains a high level of proteins [13]. Crushed, it becomes semolina, simila for the Latins or smilla in Arabic.

## 2. State Diagram of Durum Wheat Components

Durum wheat semolina is the traditional raw material for making couscous grains [3,4]. The semolina is extracted by milling kernels and corresponds mainly to the starchy endosperm.

### 2.1. Physical Characteristics

**Figure 1:**
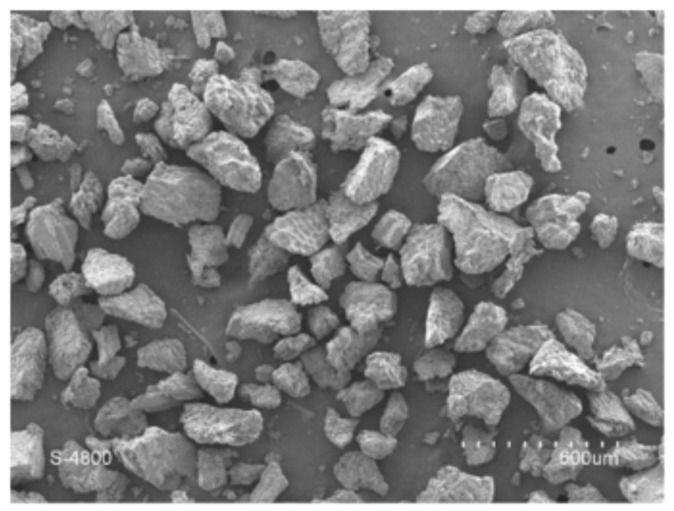
Observation of the microstructure of durum wheat semolina particles by scanning electron microscopy (reproduced from [14] with permission from Elsevier, 2022).

Durum wheat semolina is a powder with a low water content (10–14%) formed of heterogeneous and nonporous particles (Figure 1). It has a strong dispersion of diameter (between 100 and 400 µm) with a median diameter (d_50_) of nearly 300 µm.

### 2.2. Composition and Reactivity of Components 

**Figure 2:**
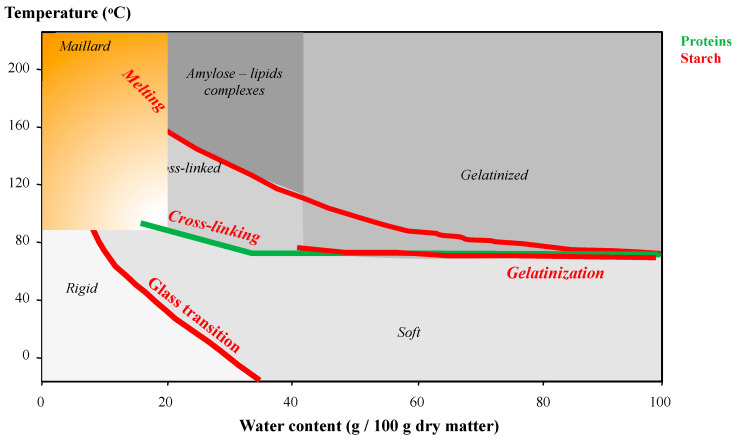
Schematic representation of transitions and physicochemical reactivity zones of wheat components (starch and proteins) according to temperature and water content conditions (adapted from [3,15] with permission from Elsevier, 2022, adapted from [4] with permission from authors).

The two main components of durum wheat semolina are starch and proteins (Table 1), with low quantities of lipids, fibers and minerals [3,4]. Physicochemical properties of starch and proteins can be described using a state diagram displaying their physicochemical reactivity as a function of temperature and water content (Figure 2) [3,4,15].

Starch is the major component (70–75%) of durum wheat semolina. Starch is an assembly of linear amylose chains and branched amylopectin chains that interact through H-bonds. In native state, starch molecules are grouped together in individualized spherical starch granules (diameters between 1 and 20 µm). In starch granules, macromolecules are assembled in concentric layers alternately crystalline and amorphous. For couscous manufacture, starch is mainly involved through its functional properties of water absorption and gelatinization, which are expressed during processes at temperatures above 50–60 °C and water contents above 40% (Figure 2) [3,4]. During couscous processing, gelatinization of starch is described as the disappearance of crystalline structures and the partial release of some amylose chains. The application of heat treatments above 100 °C could induce the formation of noncovalent interactions between released amylose chains and lipids present that lead to the formation of amylose–lipid complexes (Figure 2).

Wheat proteins represent 10–14% of semolina and are structured in amorphous individualized fibrillar form located around the starch granules. Proteins are stabilized by the presence of a high density of low-energy bonds (mainly H-bonds and hydrophobic interactions) and some covalent disulfide bonds. At temperatures above 70 °C, thiol groups present on the protein chains can participate in crosslinking reactions via the formation of disulfide bridges (Figure 2) [3,4]. Some proteins (e.g., alpha amylases, lipoxygenase, polyphenol oxidases) display enzymatic activity which can have technological consequences on couscous.

The Maillard reaction can occur between the free amine function of a protein and the carbonyl group of a reducing function of sugars. The Maillard reaction is mainly observed under conditions of high temperature (>80 °C) and low water content (<18%) (Figure 2) and leads to volatile and/or colored compounds.

### 2.3. Plasticization and State Diagram of Components

Changes in structure and reactivity of the main wheat components have been described as a function of temperature and water content on their state diagram (Figure 2) [3,4,15]. In native semolina at 20 °C and 12% water content, the structure of macromolecules is stabilized by a high density of H-bonds, in amorphous (starch and proteins) and/or crystalline (starch) states. The high density of H-bonds contributes to their low mobility and low availability to participate in reactions.

The plasticization is associated with the sensitivity of H-bonds to temperature or water content changes [16]. Thermal plasticization describes the decrease in the density of H-bonds between macromolecules due to an increase in temperature. Molecular plasticization describes the ability of added water molecules to establish H-bonds with hydrophilic groups of macromolecules, which induces an overall decrease in interaction density between macromolecules. During the processing of native semolina, small increases in temperature and/or water content thus reduce the density of H-bonds, allowing local movements of small amplitude, involving localized groups of macromolecule chains [3,4,16]. These moderate changes are observed up to a sharp transition zone, which corresponds to the expression of cooperative phenomena generating large amplitude movements involving the entire chains of macromolecules. This abrupt transition zone is called “glass transition” for amorphous structures and “melting” for crystalline structures. The construction of the state diagram of durum wheat components as a function of temperature and water content helps to locate their transition zones and different reactivity areas.

The glass transition of amorphous structures separates two states [3,4,15,16]. Below the glass transition (i.e., at low temperature and/or water content), the high density of H-bonds describes the “glassy” state: macromolecules are poorly mobile and not available to participate in reactions. Above the glass transition (i.e., at high temperature and/or water content), the low density of H-bonds describes the “rubbery” state: macromolecules are mobile and available to participate in reactions. The glass transition zone is classically described by a temperature range at a given water content. The decrease in the glass transition temperature under an increase in the water content reflects the equivalence of thermal and molecular plasticization. In durum wheat, the glass transition affects the amorphous structures of proteins and starch chains within granules. Above the glass transition at temperatures above 80 °C, proteins are available to participate in crosslinking reactions. Above the glass transition at room temperatures, an increase in water content can activate enzyme activities.

For starch, melting of crystalline structures occurs at high temperatures or water contents, above the glass transition of amorphous structures (Figure 2) [3,4,16]. Melting is classically described by a temperature range, called the melting temperature. As for the glass transition, the decrease in the melting temperature under the effect of an increase in water content reflects the equivalence of thermal and molecular plasticization. In the presence of high amounts of water, starch melting is classically associated with gelatinization. Gelatinization occurs at temperatures as low as 60 °C in the presence of high amounts of water.

## 3. Process Diagram and Structuring Mechanisms

**Figure 3:**
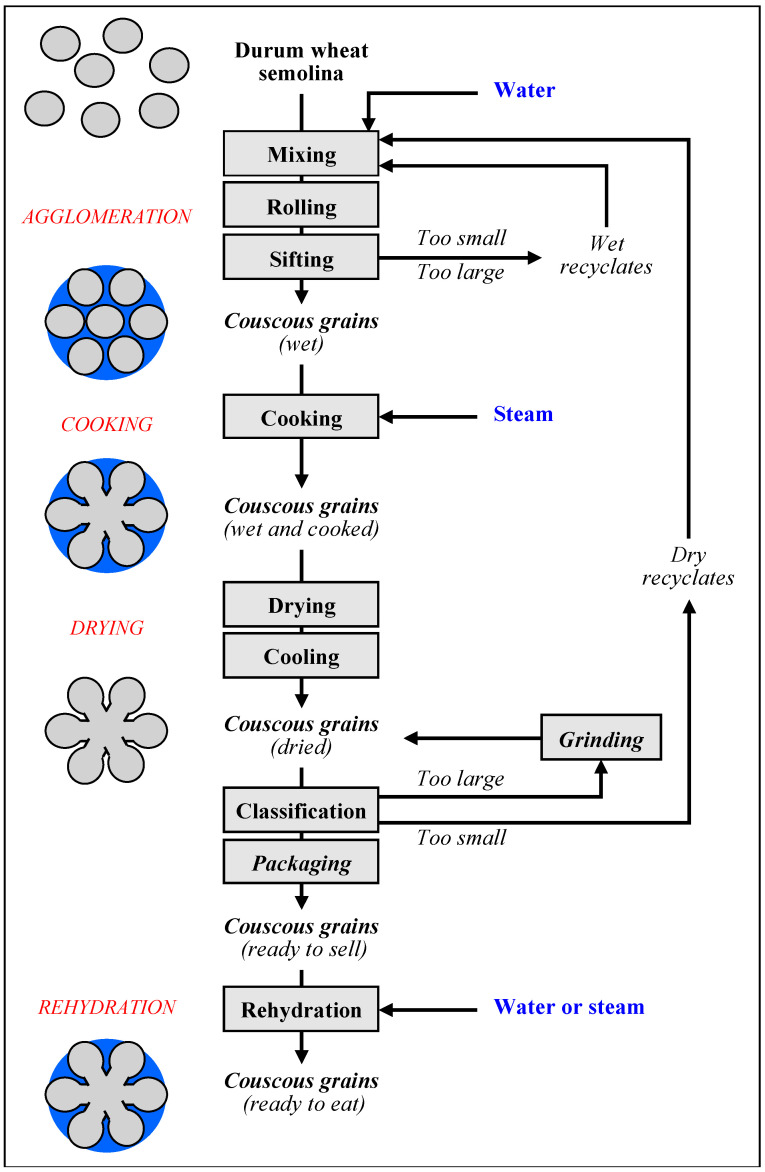
Production diagram and structural model for the processing of couscous grains from durum wheat semolina (adapted from [3] with permission from Elsevier, 2022, adapted from [4] with permission from authors).

Couscous grains are made from durum wheat semolina according to successive unit operations. A structural model to describe the transformation of durum wheat semolina particles into couscous grains is proposed by considering four phases (Figure 3) [3,4].

- *Phase 1*: Native semolina particles are agglomerated by water addition and mixing to generate the granular structure of couscous grains. The agglomeration stage is followed by a size classification stage to select grains that meet granulometric specifications and to isolate too small or too large grains, which will be recycled.
- *Phase 2*: Wet grains are consolidated by steam treatment to strengthen the internal structure through starch gelatinization, crosslinking of proteins and formation of amylose–lipid complexes. The components form a glue between semolina particles.
- *Phase 3*: Cooked grains are dried to eliminate a large part of the water, in order to ensure physicochemical and microbiological stability of the couscous grains, by reducing the water activity to about 0.5.
- *Phase 4*: Dried couscous grains have to be rehydrated before consumption. Rehydration can be completed by cold water addition, by steaming or by immersion in hot water. This step is essential to give couscous grains the firm, melt-in-the-mouth texture.

**Figure 4:**
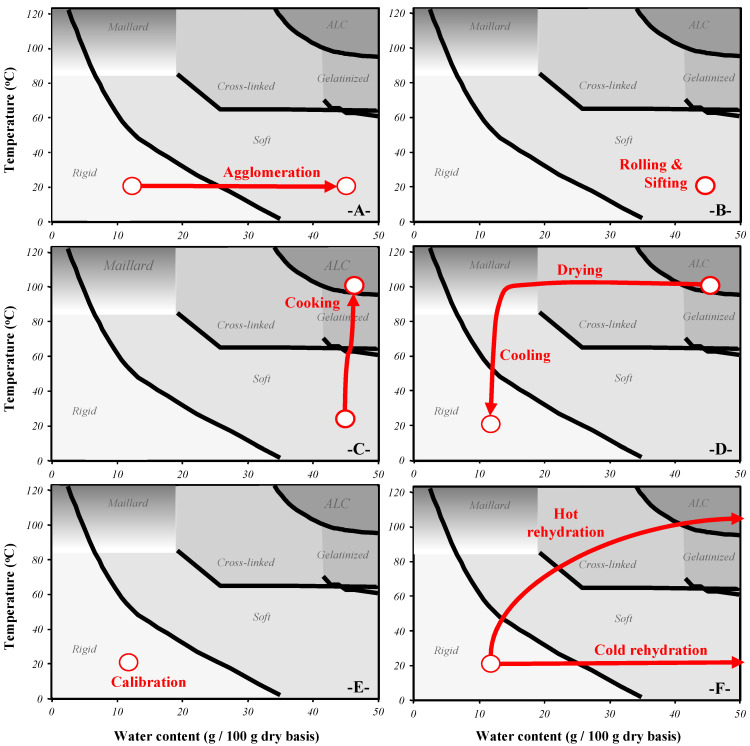
Diagram of hydrothermal paths for each unit operation of the couscous grain process on the state diagram of durum wheat components (ALC represents amylose–lipid complexes): (**A**) agglomeration stage; (**B**) rolling and sifting stage; (**C**) cooking stage; (**D**) drying and cooling stage; (**E**) calibration stag; (**F**) rehydration stage (adapted from [3] with permission from Elsevier, 2022, adapted from [4] with permission from authors).

The description of the couscous grain process on the state diagram of durum wheat components makes it possible to link changes in the process parameters for each unit operation with induced mechanisms (Figure 4) [3,4].

### 3.1. Agglomeration

**Figure 5:**
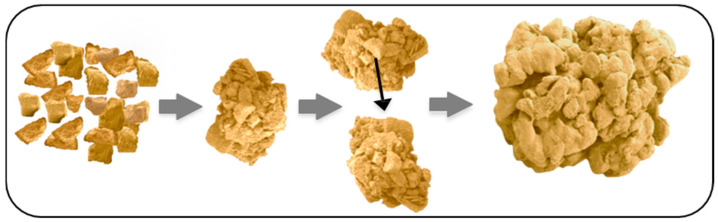
Schematic representation of wet agglomeration mechanisms of durum wheat semolina for the generation of agglomerates (adapted from [4] with permission from authors).

The agglomeration of semolina is induced by the simultaneous addition of water (up to a water content of about 45%) and mixing, at constant temperature around 20–25 °C. Agglomeration mechanisms correspond to the structuring by the assembly of small semolina particles to form larger agglomerates. A necessary and sufficient amount of water must be added to induce the formation of cohesive contacts between native particles, generate a granular structure and ensure the internal cohesion of agglomerates [3,4]. Mixing ensures the homogeneous dispersion of water, promotes contacts between hydrated particles and generates growth mechanisms (Figure 5). Hydration properties of semolina and mechanisms of wet agglomeration by wetting and mixing have been studied in several works [6,14,17,18,19,20,21,22,23,24]. The multiplicity of mechanisms identified during the agglomeration of the semolina controls the characteristics of couscous grains [3,4].

The hydration and mixing stage plays an essential role in agglomeration mechanisms of semolina particles, generating different heterogeneous granular structures [3,4,18]:

- Fine particles (diameter < 0.5 mm) are residual particles of native semolina, or small wet particles generated by erosion mechanisms of larger agglomerates.
- Wet nuclei (0.5 < diameter < 0.8 mm).
- Wet agglomerates (0.8 < diameter < 2 µm) are the desired structures and will give the couscous grains.
- Large pieces of “dough” (2 mm < diameter).

Physicochemical mechanisms: The addition of water induces the passage of the glass transition of amorphous zones of starch and proteins (Figure 4A), which allows enzymatic reactions involving peroxidases and polyphenol oxidases in semolina [25]. During couscous processing, a significant decrease in carotenoid pigment content was observed during kneading and rolling stages [26]. Even if starch and proteins are not directly involved in agglomeration mechanisms, the passage of the glass transition allows macromolecules to participate in adhesion and sticking mechanisms [27,28,29,30]. The agglomeration stage results in a decrease in the insoluble glutenin content and an increase in the soluble glutenin and SDS-soluble protein contents. These mechanisms were attributed to the dissociation of large SDS-insoluble glutenin polymers due to the shearing effects of large dough pieces during mixing. Based on electrophoresis, Lefkir [30] indicated that the agglomeration stage of semolina does not allow the development of continuous protein networks. The amount of added water and mechanical energy inputs are not sufficient to induce the formation of a gluten network.

Agglomeration mechanisms are impacted by the particle size distribution of semolina [30,31]. The decrease in the median diameter of semolina leads to an increase in the proportion of agglomerates and a decrease in the proportion of dough pieces and small particles. Using semolina with a low diameter span reduces the proportion of small particles after agglomeration. Conversely, the protein content of semolina does not impact the proportion of wet agglomerates after mixing [31]. Lefkir [30] showed a slight positive correlation (*r* = 0.82) between the protein content of semolina and the proportion of large dough pieces after mixing. The protein content of semolina does not seem to control the agglomeration yield. Lefkir [30] found a positive correlation between the content of soluble glutenins in wet agglomerates and agglomeration yield.

The amount of added water is a key parameter as it controls the size, density and shape characteristics of wet agglomerates and couscous grains [3,4]. A sufficient amount of water is required to ensure agglomeration yields and to promote mechanisms during the subsequent cooking stage [32]. Increasing the amount of added water results in an increase in the proportion of wet agglomerates and dough pieces and in a decrease in the proportion of fine particles [18,19,21,25,33,34,35]. An increase in hydration could favor the solubilization mechanisms of glutenins [30]. A decrease in water temperature results in an increase in the proportion of agglomerates at the end of mixing, associated with a decrease in the proportion of dough pieces [34]. An increase in the water temperature is unfavorable for the solubilization of glutenins [27,30]. The mixing time has a significant impact on agglomeration mechanisms [25]. Water homogenization between different fractions was also observed during the mixing stage [36]. Long mixing times increase the proportion of fine fractions and decrease the proportions of medium and coarse fractions due to specific breakage mechanisms.

### 3.2. Rolling and Sifting

After mixing, the agglomerated powder is submitted to a combined rolling and size classification stage [3,4]. This stage is conducted at room temperature and without changes in water content (Figure 4B). The rolling operation corresponds to the movement of granular objects on the metal surface of a succession of sieves with different opening diameters. The displacement of the wet powder on sieves contributes to structuring mechanisms of the grains, with slight densification and some erosion effects on the agglomerates [37]. Rolling conditions (type of roller, agitation forces, layer thickness, duration) control the intensity of these changes. The displacement of the wet powder on the surface of sieves ensures the classification by the diameter of the wet granular objects. The size classification allows removing too small particles (diameter < 0.8 mm), recovering wet agglomerates with the target size (0.8 < diameter < 2 mm) and removing too large dough pieces (2 mm < diameter). Too small particles are recovered and reintroduced at the mixing stage to again participate in agglomeration mechanisms. Large dough pieces are shredded before being reintroduced into the mixer. Wet agglomerates with a size within the target diameter are wet couscous grains that will undergo the next cooking stage.

### 3.3. Steam Cooking

The internal structure of grains is consolidated by a steam cooking stage [3,4]. Wet grains are exposed to a stream of steam at 100 °C for a period of about 10–20 min. Steaming results in a rapid increase in the temperature of couscous grains up to 100 °C and induces a slight increase in their water content due to steam condensation and water absorption (Figure 4C). Due to their small diameter (1–2 mm) and circular shape, heat transfers in grains are not limiting factors because they are quick enough (about 1 min) to allow grains to rapidly equilibrate with the steam temperature. During steaming, the water content of grains rapidly increases from 0.48 to 0.53 g/g dry matter [32]. Water absorption by couscous grains during steaming is more important when using fine semolina as raw material [38]. During steaming, the diameter of grains increases from 1.6 to 2.15 mm due to swelling mechanisms, but this does not impact the spherical shape of the grains. The steaming induces different mechanisms involving wheat components that contribute to strengthening the structure of couscous grains [3,4].

(i) Steaming induces the gelatinization of starch granules with loss of crystalline structures and the partial release of amylose chains [3,4]. These changes participate in the formation of a sticky cement between the semolina particles. The extent of starch gelatinization increases rapidly as a function of the steaming time and reaches 80–100% [32,39]. The homogeneity of gelatinization mechanisms controls the water absorption properties of couscous grains [25]. The sticky behavior of couscous grains after rehydration could be due to the presence of amylose chains on the surface of grains or heterogeneous steaming [40].

(ii) Steaming also induces the formation of amylose-lipid complexes [3,4]. After gelatinization of starch granules and at high temperatures, released amylose chains can be available to participate in complexation reactions with monoglyceride molecules, which are present in native semolina at low contents. The extent of the complexation of amylose with lipids depends on the steaming duration [40]. The initial content of lipids would be the limiting factor for improving the culinary quality of couscous. The formation of amylose–lipid complexes strengthens grains, contributes to limiting the sticky behavior of rehydrated couscous grains and reduces the retrogradation phenomena during storage [25].

(iii) Steaming induces insolubilization of wheat proteins through the formation of covalent disulfide bonds [3,4,25,41]. Insolubilization of glutenins occurs rapidly during steaming [30,41]. Conversely, gliadins are little affected by steaming. An increase in the hydration level or a decrease in the water temperature at the mixing stage favors the insolubilization of glutenins during steaming [30]. Crosslinking reactions could contribute to decreasing the stickiness of couscous grains [3,4].

Before steaming, wet couscous grains are formed by the brittle assembly of semolina particles that adhere to each other. The steaming induces a partial melting of semolina particles which then irreversibly bind to each other. After steaming, couscous grains display a homogeneous structure with a “melted” appearance, in which semolina particles are hardly identifiable [32]. The extent of steaming greatly contributes to the water absorption, stickiness and texture properties of rehydrated couscous grains. Steaming makes the starch ingestible due to the gelatinization mechanisms. Couscous grains can be consumed directly, after a simple rehydration using cold water.

It should be noted that the steaming stage is classically called “precooking” and is followed by a “cooking” stage when preparing the couscous grains before consumption.

### 3.4. Drying and Cooling

Cooked couscous grains are dried in hot dry air to reduce their water content to a value of less than 13.5% in order to comply with the legislation [3]. The small diameter and porosity of couscous grains facilitate water removal mechanisms. A thin layer of couscous grains is exposed to dry air and/or subjected to agitation to favor water extraction. During drying, water transfers are the limiting phenomena [32]. Yüksel et al. [42] calculated effective moisture diffusivities of couscous grains (between 1 × 10^−8^ and 1.7 × 10^−8^ m^2^·s^−1^) according to Fick’s second law for sphere geometry in one dimension. The drying curves of a packed couscous bed were linear with a constant drying rate. Increasing air temperature (from 60 to 80 °C) led to an increase in effective diffusivity and drying rates. The extraction of water during drying induces shrinkage of grain structure: the volume of water extracted from the product is almost compensated by the volume contraction of grains and the reduction in diameter [32]. The shrinkage of grains during the drying stage is possible due to the plasticization of wheat components above the glass transition (Figure 4D). When the drying stage is conducted at high temperatures (90–120 °C), complementary mechanisms of glutenin insolubilization and amylose–lipid complex formation can be observed [30]. The drying stage contributes to the strengthening of couscous grains. Drying at high temperatures favors the Maillard reactions, especially at the end when the water activity is low. These reactions can lead to the formation of brown-colored compounds and specific volatile compounds.

After drying, the cooling phase until ambient temperature allows the rigidification of couscous grains thanks to the passage under the glass transition curve of amorphous wheat components which become rigid [3].

### 3.5. Calibration by Size

After drying, it is necessary to size-grade dried grains on a vibrating sieve column to recover couscous grains and to separate too fine or too large particles [3]. The classification stage is carried out at room temperature and does not significantly change the water content of couscous grains (Figure 4E). Thanks to the size classification stage, the granulometric dispersion of dried couscous grains is relatively low.

### 3.6. Storage

Dried couscous grains are packed in the appropriate packaging. Only little work describes the behavior of couscous grains during storage. The lipid fraction is critical during couscous storage, through oxidation mechanisms and the appearance of rancidity off-flavors [43]. These mechanisms can be reduced by using a high-temperature drying cycle which can be more effective to inactivate lipase. Guezlane et al. [40] showed that gelatinized starch in couscous grains is not sensitive to retrogradation phenomena during storage, because structures of the amylose–lipid complexes remain present.

### 3.7. Rehydration before Consumption

Before being consumed, couscous grains must undergo a final rehydration stage [4]. Depending on the intended use, dry couscous grains can be rehydrated by mixing with water at room temperature, by mixing with hot water or by exposure to steam (Figure 4F). For conventional uses, it is recommended to hydrate the couscous by mixing similar volumes of water and couscous to reach a final water content close to 60%. The increase in water content ensures the glassy transition of the wheat components into the rubbery domain, which contributes fully to the smooth texture of the ready-to-eat couscous grains.

## 4. Domestic Production of Couscous

**Figure 6:**
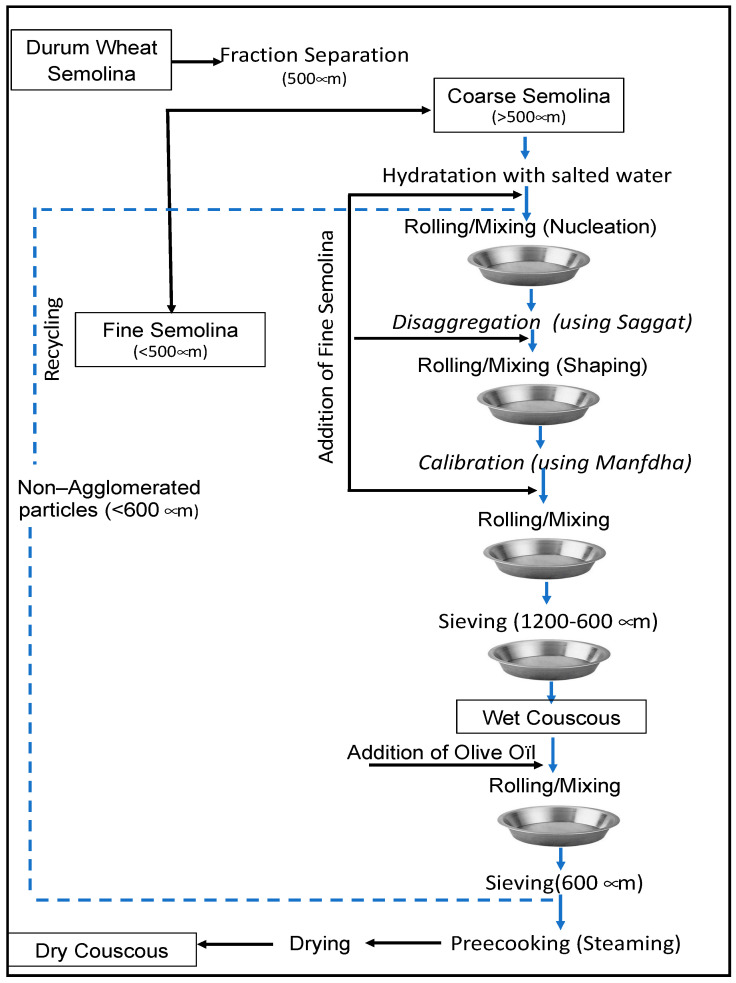
Traditional couscous making diagram identified from the handmade process used in Northern Tunisia.

The manufacture of artisanal or domestic couscous is carried out in summer (from May to September) at home in a clean and well-ventilated room [5]. Experienced women are dedicated to manufacturing couscous following successive steps (Figure 6). Traditional couscous production requires a large workforce. The process involves mixing water and durum wheat semolina in a large wooden dish and then rubbing the mixture between the palms of the hands to form agglomerates or small irregularly shaped granules. Granules are then separated by a set of appropriate sieves, and the desired portion is retained. The control of the agglomeration and hydration processes is very important to produce couscous with desired quality. In Maghreb countries, processes of making artisanal couscous differ from one region to another or even from one person to another. Details concerning the ethnic preparation of couscous in Tunisia are presented in the following sections.

### 4.1. Utensils

**Figure 7:**
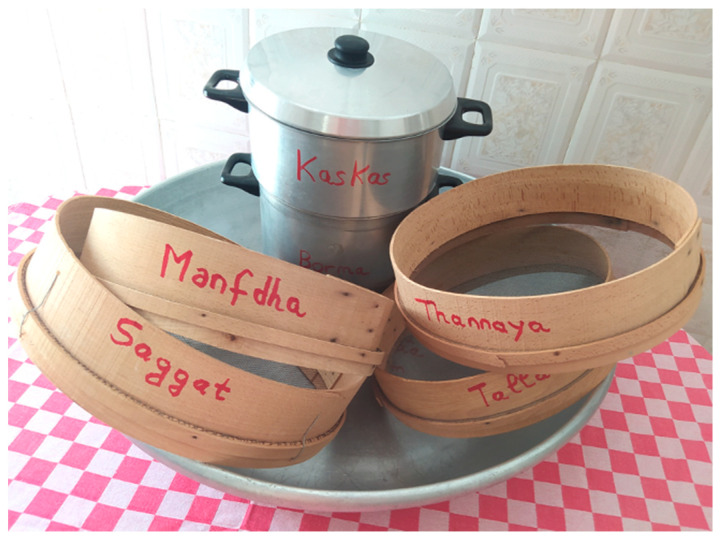
The utensils used for the domestic manufacture of the couscous in Tunisia.

**Figure 8:**
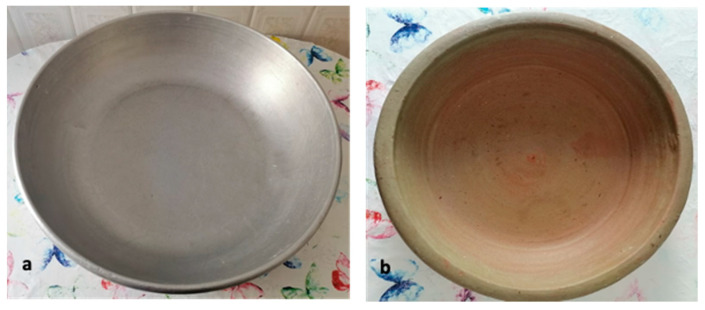
“*Guassâa*” made from aluminum (**a**) and tierra clay (**b**).

**Figure 9:**
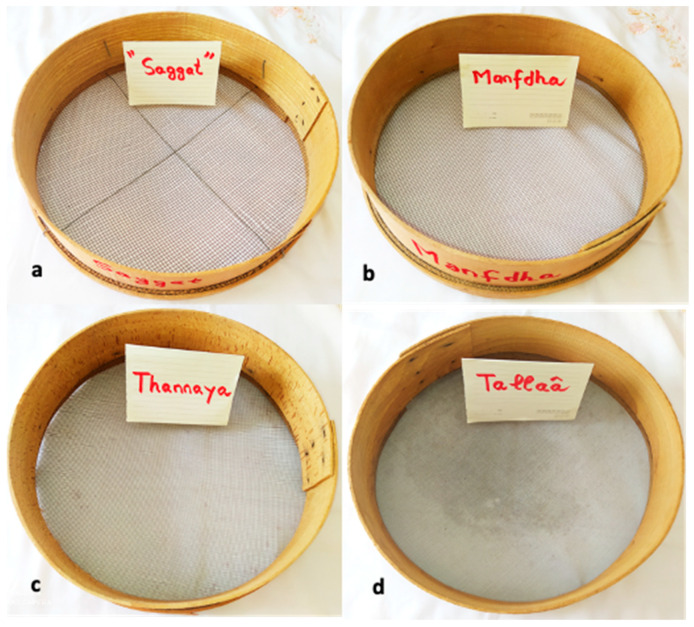
Different sieves used for semolina calibration and couscous shaping: (**a**) “*saggat*” used for lump disaggregation; (**b**) “*manfdha*” used for couscous calibration; (**c**) “*thannaya*” used 2 or 3 times for couscous sieving; (**d**) “*tallâa*” used for semolina sieving and finishing.

**Figure 10:**
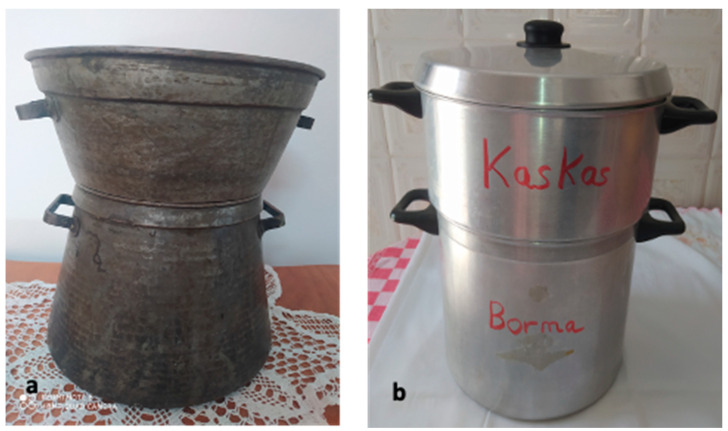
Examples of “*couscoussiers*”, traditional steamers for preparing couscous, that are made of copper (**a**) or metal (aluminum) (**b**).

In Maghreb countries, couscous is still prepared manually at home using different utensils [5] (Figure 7). The humidification and rolling of the semolina are carried out on the “*guassâa*”, a wide bowl and hollow plate in wood or aluminum clay (Figure 8). Sizing and calibration of wet grains are performed on different sieves named “*saggat*”, “*manfdha*”, “*thannaya*” and “*tallâa*” (Figure 9), depending on mesh opening (2.3, 1.2, 1 and 0.6 mm, respectively). The traditional double-chambered food steamer is used to cook couscous by North Africans and now worldwide. This utensil is called “*couscoussier*” in French (“*taseksut*” in Berber language). It is made from ceramic or metal and consists of an upper smaller pot (“*kaskes*” in Arabic) containing holes that allow the passage of steam (Figure 10). The lower part is a large pot (“*borma*” in Arabic) that holds the meat and vegetables to be cooked as a stew in water or soup and produces steam. Once the couscous is steamed, the lower pot is kept at a simmer until cooking is complete [6].

### 4.2. Preparation and Classification of Semolina

**Figure 11:**
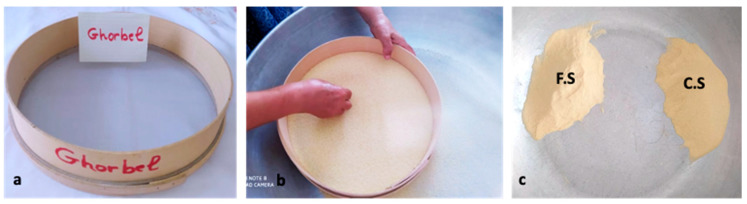
Semolina classification: (**a**) sieve used for classifying semolina known as “*ghorbel*” in Arabic and “*agherval*” in Berber language; (**b**) semolina classification; (**c**) semolina separated into 2 fractions: coarse semolina (CS) and fine semolina (FS).

Homemade couscous is classically prepared from coarse semolina. Semolina classification is performed to separate two fractions by using a 0.5 mm mesh opening sieve (named “*ghorbel chaâr*”): coarse semolina (named “*fetla*”) and fine semolina (named “*dkak*”) (Figure 11). Classification improves the agglomeration yield of semolina by allowing the formation of agglomerates rather than clumps of dough. In Tunisia, making artisanal couscous obeys the classification step, but coarse semolina is used directly without sieving. The survey conducted by Chemache et al. [6] indicated that 20% of interviewed women state that the classification operation is not necessary if there is homogeneity in the particle size distribution of the semolina.

### 4.3. Hydration and Mixing

**Figure 12:**
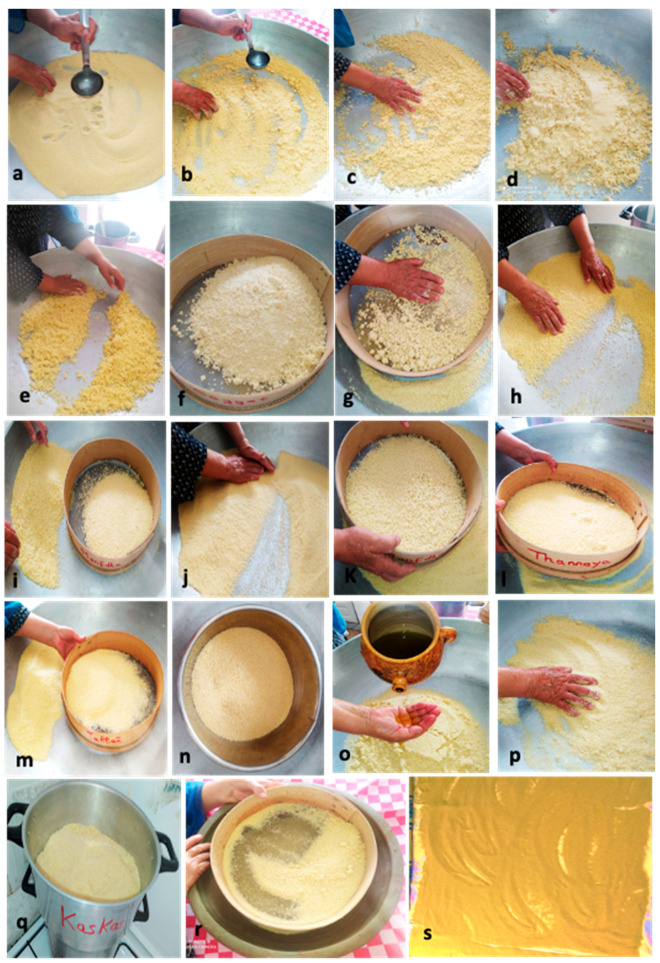
Overview of the traditional handmade couscous according to the method used in Northern Tunisia: (**a**,**b**) semolina hydration with salted water; (**c**) rolling mixing of coarse semolina and salted water; (**d**) adding a small quantity of fine semolina to avoid over-agglomeration; (**e**) rolling mixing; (**f**,**g**) breaking down lumps with “saggat”; (**h**) rolling mixing; (**i**–**m**) sieving using different kinds of opening mesh sieves; (**n**) wet couscous; (**o**) adding a small quantity of olive oil; (**p**) rolling mixing; (**q**) steaming; (**r**) sieving using “*thannaya*”; (**s**) drying.

The wet agglomeration is usually carried out in the “*guassâa*” (Figure 12a,b). This requires the double effect of mixing and adding salted cold water on coarse semolina which allows particle sticking. This critical step leads to the agglomeration of semolina to form couscous grains [35]. It is very important to obtain a homogeneous wetting of the semolina and to ensure the wetting of the semolina formation of dough pieces that will make the rolling operation difficult. Cold water helps to avoid the formation of large agglomerates. According to a survey [6], salt is added (salt content is 1.6%) to enhance the flavor of the final product. The characteristics of obtained wet agglomerates contribute greatly to the final couscous grain quality [35].

### 4.4. Rolling and Calibration

Rolling is the operation of shaping couscous by agglomeration of the hydrated semolina particles. This operation is conducted in four main substeps known as nucleation, shaping, sieving and finishing.

(a) Nucleation: The rolling process begins with simultaneous watering and mixing of both semolina fractions. First, the watering is performed gradually with small volumes of salted water using a ladle or by hand (Figure 12b). Second, the whole is mixed in circular movements by hand fingers half bent to distribute the wetting liquid in the bed powder in a homogeneous way (Figure 12c). The addition of small quantities of fine semolina allows the initiation of particle nucleation (Figure 12d). The wetting liquid is absorbed by the fine particles, which serve as nuclei around which coarse particles adhere [6,33,34]. The most influential parameter on the rolling yield is the semolina hydration rate [25]. The rolling operation is easier with coarse semolina [34].

(b) Shaping: Primary grains formed during the hydration step are grown by the addition of fine semolina [6]. At this stage, rolling is carried out by applying energetic and circular movements with the palm on the particle bed (Figure 12e). The fine semolina aggregates onto primary grains (nuclei). This step allows the formation of larger agglomerates through a snowball effect and coalescence. The fine semolina adheres against the voids of grains and gives spherical and smooth agglomerates. According to a survey [6], the rolling operation of semolina is carried out two or three times to ensure that it has absorbed all the added water. This stage allows good cohesion between semolina particles. In this step, lumps are broken down through a mesh sieve (called “*saggat*”) (Figure 12f,g). If there is an exaggerated agglomeration, a small quantity of fine semolina could be added, and lumps are broken down using the “*saggat*” two or three times.

(c) Sieving: The sieving operation corresponds to setting in motion the agglomerates on the surface of a succession of sieves of decreasing mesh to ensure a classification by size. The sieving step is important to obtain the desired homogeneity and size of the couscous. Agglomerates formed during the previous step are broken down through successive sieves (called “*manfdha*”, “*thannaya*” and “*tallâa*”) (Figure 12i,l,m). The under-size fraction will undergo rolling several times before calibration or sieving (Figure 12n). The recycling process is repeated until maximum depletion of semolina, but it is impossible to obtain a rolling yield of 100%. The grains with a size greater than 0.6 mm undergo a finishing step [5,6].

(d) Finishing: This step consists of a rolling operation of wet couscous grains using a small quantity of olive oil. Women add a small quantity of olive oil and perform a circular movement several times (Figure 12o). Olive oil is used to homogenize and improve the texture of couscous grains by giving them a more spherical shape and a smooth surface and producing well-individualized grains (Figure 12p). Chemache et al. [6] reported that instead of olive oil, Algerian women also use wheat flour or corn starch and obtain similar results.

### 4.5. Steaming

The finished wet couscous of the desired particle size is put in the upper part of the “*couscoussier*” containing boiled water. Couscous is steamed for 15 min (Figure 12q). Steaming time depends on the thickness of the couscous layer and on the couscous granulometry: large couscous grains require a shorter cooking time as the water vapor circulates more rapidly between coarser grains [44]. The precooking time is determined when steam is on the surface of couscous. Cooked couscous grains break apart between fingers in the form of dough and have a yellow color. Immediately after steaming, the manual lump breaking (Figure 12r) is carried out using the “*thannaya*” sieve to obtain separated cooked grains, ready for drying. In some regions of Tunisia, couscous does not undergo drying. However, before serving, it has to be steamed two or three times. The “moist couscous” is prepared in the same way as the dry couscous (except the drying stage) and is prepared and consumed the same day [45].

During the traditional steaming, the couscous can be subjected to two successive cooking stages interrupted by the addition of fats (butter or olive oil) [40,46]. Amylose chains released by the starch gelatinization during the first steaming stage can form complexes with added lipids, in addition to those present in the native semolina. Enhancing the formation of amylose–lipid complexes reduces stickiness, delamination and caking index and increases the firmness of couscous grains upon rehydration [25,46,47,48,49,50]. The impact on organoleptic characteristics of couscous depends on the type of lipids used.

### 4.6. Drying

The drying stage of couscous is conducted in two phases. The couscous is first spread out on a clean sheet in the shade at ambient temperature (Figure 12s) for a duration depending on the air temperature and relative humidity. This first phase allows the preservation of couscous qualities. When the couscous is “sufficiently” dried, the couscous is then dried in the sun to ensure optimum water elimination. Couscous is occasionally stirred for a good drying process. The drying step is strictly related to climatic conditions that account for the production of home-made couscous during the sunny summer months [12]. Sun-dried couscous has a long shelf life.

### 4.7. Grading

The couscous is separated into fine, medium and coarse. The final product is classified in three different sizes: small couscous (diameters < 1.5 mm) is recommended for desert preparation; medium couscous (1.7 < diameter < 2.0 mm) is the most appreciated for traditional dishes; coarse couscous (2.5 mm < diameter) is used to prepare couscous with vegetable sauce [12].

### 4.8. Storage

**Figure 13:**
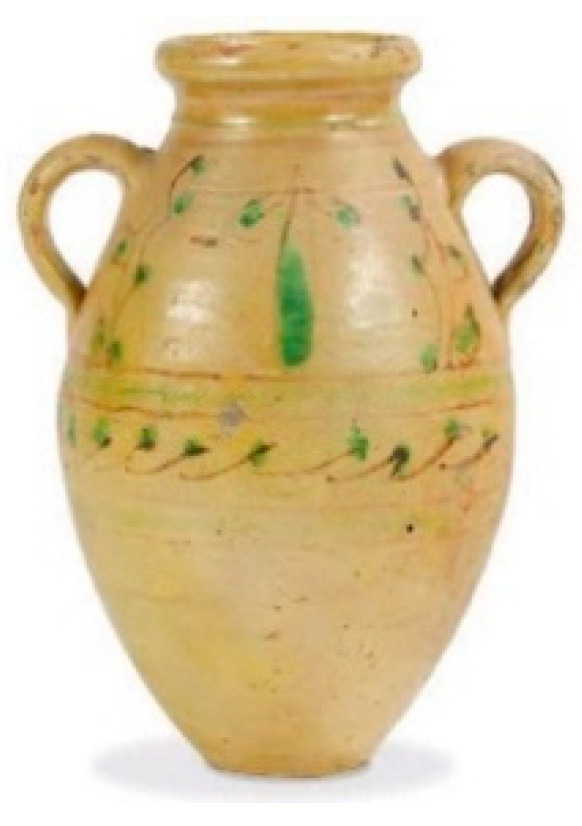
Picture of a jar named “*khabiya*” in Arabic language used to store couscous.

The couscous is stored until use in cloth bags or in a large jar named a “*khabiya*” (Figure 13) and kept in a dry place at room temperature. To enhance the shelf life or to improve the organoleptic qualities of couscous, homemakers can add ingredients such as black or red dried pepper and bay leaf [6].

### 4.9. Couscous Dish Preparation

**Figure 14:**
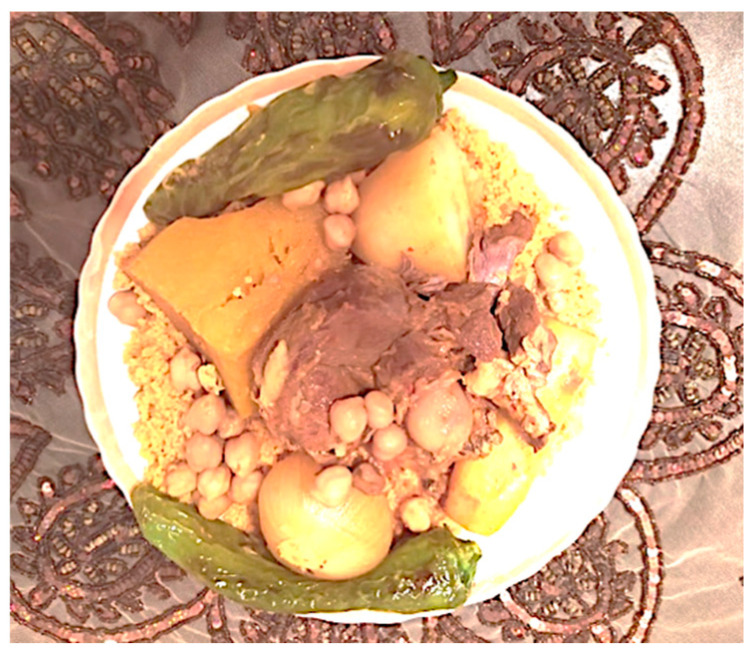
Picture showing the typical dish of couscous prepared with meat and vegetables.

The final step of rehydration before consumption can be carried out using cold or warm water. The dry couscous is soaked in warm water for a few minutes, followed by draining in a couscous pot (Figure 14). Afterward, the rehydrated couscous is immediately drained, allowed to stand for about 8–10 min, stirred and dispersed from time to time before the rehydrated couscous is added with the fat. Several types of fat can be used, such as olive oil. The choice of added fats is based on their availability and consumption at family events. Melted butter (“*dehane*”) is the most preferred when preparing couscous to be served during celebrations. The hydrated couscous is put in the couscous pot “*kaskas*” that is placed over a pot containing the sauce being cooked. Several criteria have been listed to stop the final cooking: the rise of the vapor, the development of the bright yellow color and an increase in the volume of cooked couscous grains [6]. Subsequently, the couscous is crumbled and watered with a small amount of water. Couscous can be served in many different ways and with a variety of foods.

## 5. Industrial Production of Couscous

The first industrial production of couscous was initiated in North Africa in the 1960s and later in France, Italy, Greece and the United States [3,4,5,39,43]. Although the consumption of couscous is worldwide, the manufacturing industry is still mainly located around the Mediterranean. The first pieces of industrial equipment were simple transpositions of the lines used for the production of short pasta. From the 1970s, fully automated couscous production lines were developed. The design of equipment sought to reproduce the gestures mastered for the artisanal manufacture, particularly the agglomeration and rolling stages, in order to obtain qualities similar to domestic couscous. For the past 20 years, equipment manufacturers have been offering industrial lines that are specifically adapted to optimize industrial performance and product quality, with flow rates reaching 500 to 1500 kg·h^−1^. The specificity of the couscous manufacturing process is the management of the homogeneous treatment of granular materials, from semolina to couscous grains.

### 5.1. Specifications for Durum Wheat Semolina

Only a few scientific works have investigated the contribution of the characteristics of semolina to the process behavior and qualities of couscous grains. Originally, specifications for semolina for couscous were similar to those for pasta [3,4,5,12,38,43,51,52]. However, the use of high-quality semolina is not a requirement for the production of couscous. It is classically recommended to use durum wheat semolina with coarse diameter, as the size of semolina plays a role in defining the process settings [3,5]. Because of its higher water absorption, coarse semolina requires less water during mixing and results in a higher couscous yield than fine semolina [3].

The characteristics and content of wheat components (proteins, ash, gluten index, damaged starch, etc.) of semolina only play secondary roles in structuring mechanisms and qualities of couscous [3,5,43,53]. The role of semolina protein content remains unclear [5]. On one hand, Boudreau et al. [51] indicated that the couscous value of semolina depends on its protein content, close to 13.5% being preferred. Debbouz et al. [33] observed that wheat varieties with strong gluten expressed a better yield of couscous than cultivars with weak gluten. They referred to a decrease in stickiness as protein content increased. On the other hand, Ounane et al. [49] demonstrated that the semolina protein content, dry gluten contents and gluten index were poorly related to couscous characteristics. Concerning lipids, no correlation was found between the semolina total lipid content and cooked couscous quality [49]. Conversely, contents of apolar lipids, polar lipids and polar bound lipids of semolina could affect couscous qualities.

### 5.2. Hydration and Mixing

On an industrial scale, two types of equipment can be used for the agglomeration stage with wetting and mixing unit operations [3,4].

(i) Equipment based on the simultaneous water addition and mixing in a horizontal mechanical mixer with two rotating axes [3,4]. This process mimics traditional gestures and practices while intensifying the technique. Water is supplied by flowing directly onto the semolina during mixing. The high rotation speed of mixing shafts is required to ensure the homogeneous distribution of the water within the semolina and to generate agglomeration mechanisms.

(ii) Equipment based on individual hydration of the particles before mixing [3,4]. The semolina particles are individually hydrated by spraying water in a mechanically intensive system with a high speed of rotation of a mixing shaft to individualize the particles. This system generates water droplets to be evenly distributed over each particle of semolina. Intensive wetting is immediately followed by intense mechanical mixing in a double-axis horizontal mixer to promote agglomeration mechanisms.

The management of the agglomeration stage is critical as it determines the performance of the production lines. The agglomeration stage can generate large amounts of by-products after the rolling stage (i.e., too small or too large particles), which can represent a mass flow up to 2.5 times greater than the flow of the native semolina [3,4]. Minimizing these flows has obvious implications because of the unnecessary energy expended to reincorporate these products and the oversized equipment for mixing and classification.

### 5.3. Rolling and Sifting

Two types of equipment are available for carrying out the rolling and sifting operations [3,4].

(i) The plansichters consist of a series of superimposed flat vibrating sieves with openings of decreasing diameter. They are used to replicate the manual gestures. The too large particles are retained on the first sieve. Wet agglomerates with a size within the target diameter are retained and collected on the second sieve. Fine particles pass through the second sieve. The rolling operation on vibrating sieves significantly impacts the density and shape of wet agglomerates [54].

(ii) The rotary drum rollers consist of a succession of sections within a slightly inclined cylindrical drum. The first section is formed by unperforated metal plates. The following sections consist of a succession of perforated plates with holes of increasing diameter. The wet granular material is introduced at the inlet of the drum. The drum rotation helps to advance the granular material. Some mechanical effects are generated by the flow of the granular bed induced by the rotation of the drum. Too fine particles are removed at the first screens. Couscous grains are collected at the next grids. Coarse particles flow to the end of the drum. Couscous grains rolled in a rotating drum are more spherical and denser than grains rolled on plansichters [3,4,54]. The rotating screen drum parameters (angle of inclination, rotating speed and product flow rate) do not impact the sieving efficiency and characteristics of the agglomerates (diameter, water content and porosity), as no secondary agglomeration phenomena significantly occur [37].

### 5.4. Steam Cooking

Wet couscous grains are cooked by steam injection in a continuous tunnel cooker [3,4]. Grains are deposited to form a thick layer of about 20 cm on a perforated metal belt that passes through the tunnel. Steaming is carried out at 100 °C by injecting steam at atmospheric pressure through the product layer. A residence time of 15–20 min is required to ensure steam flow and heat transfer within the product layer. Cooking a thick layer of couscous can result in heterogeneities in the distribution of cooking values, especially between the surface and the core of the couscous bed, or in the case of heterogeneous steam circulation within the product layer. These heterogeneities can result in undercooked or overcooked couscous grains [3,4]. Some industrial lines use steam injectors to spray steam over and under the product, assuring a more homogeneous cooking [43]. At the cooked exit, the layer of cooked couscous grains forms a sort of cohesive “cake” that must be separated mechanically using a specific mixer combined with a calibration sieve to individualize cooked couscous grains before the drying stage.

### 5.5. Drying

The drying stage of couscous grains is conducted on pods circulating in a hot air drying tunnel, with controlled flow rate, temperature and relative humidity of air [3,4]. Industrial drying of couscous is carried out at high temperatures (90–120 °C) over short periods of time (15–20 min). The movement of the pods allows a “soft” mixing of products to favor mass exchanges with the hot air stream, limiting the formation of a static layer barrier to transfers. The movement of the pods can lead to erosion of grains, resulting in the formation of “fine dried particles”. After the drying stage, dry and hot couscous grains are cooled to room temperature in a cooler with a cold air stream [3,4].

### 5.6. Sifting

Dry couscous grains are graded according to size criteria depending on the target diameter [3,4]. Products collected at the exit of the cooler are deposited at the top of a column of vibrating sieves, with decreasing mesh. The products are separated into three categories.

- Too fine particles with a diameter below the target diameter mainly come from breakage or erosion mechanisms of couscous grains during the drying stage. The flow of fine particles can represent 5–7% of the throughput of dry couscous grains.
- Several dry couscous grains in the diameter target can be produced: fine (0.63 < diameter < 1.25 mm), medium (1.25 < diameter < 1.85 mm) or coarse (1.85 < diameter < 2 mm) couscous grains.
- Too large particles with a size greater than the target diameter are usually clusters of several couscous grains that have stuck together during the cooking or drying stages. These particles are sent to a roller mill for size reduction and then sifted again.

### 5.7. Recycling Discarded Products

The classification (after rolling) and calibration (after drying) stages discard significant flows of too small or too large products [3,4]. These products are characterized by a composition similar to native semolina, but with specific values of the extent of starch gelatinization and the solubility of proteins (Table 1).

- The dried products discarded after the drying stage display biochemical characteristics equivalent to dry couscous grains, with a high extent of starch gelatinization and a low solubility of the proteins. They are sent to a specific hydration stage before mixing with native durum wheat semolina [3,4].
- The products discarded after the rolling stage display physicochemical characteristics similar to native semolina. The main difference is a higher water content. These products are characterized by a slightly higher gelatinization extent (about 10%) and lower protein solubility than native semolina [3,4]. These differences are not due to the agglomeration and rolling stages. They are mainly due to the flow of dry discarded products which are reincorporated at the agglomeration stage with the native semolina. The extent of starch gelatinization is consistent with the reincorporation ratio (5–7%) of the fine dried particles [3,4].

Discarded products are reintroduced at the agglomeration stage (Figure 3). The differences in the physicochemical state of the components of the discarded products (Table 1) result in differences in water absorption capacity and physicochemical reactivity compared to the native semolina [3,4]. The contribution of discarded products to the structuring mechanisms of couscous grains is still little known. The reincorporation of discarded products requires specific know-how to adjust parameters, namely the amount of added water and the reincorporation ratio, at the agglomeration stage.

### 5.8. Rehydration before Consumption

The diversity of marketing channels for couscous grains generates great diversity in rehydration methods, whether by companies preparing tabbouleh-type dishes, by catering companies using couscous grains for cold or hot preparations or by individuals. Several methods of rehydrating couscous grains can be described [4]:

- To prepare the tabbouleh, rehydration by adding an equivalent volume of tap water, mixing and resting for 30 to 60 min.
- For the traditional preparation of couscous, rehydration by contact with a steam flow for a defined period of time.
- For rapid hot preparation, there are several possibilities for rehydration.
- By adding an equivalent volume of boiling water, mixing and resting.
- By immersing a “cooking” perforated plastic bag in excess water for a defined period of time and draining.
- By mixing with an equivalent volume of cold water and heating in a microwave oven for a defined time.

## 6. Characteristics of Couscous

Over the past 30 years, as research has progressed and needs have arisen, a set of specific analytical methods has been developed to evaluate the qualities of dried couscous grains and of couscous grains after rehydration [3,4,12,22,25,33,39,43,49,55]. The evaluation is based on visual, usage and organoleptic criteria using instrumental or sensory methods. Couscous grain quality depends on the characteristics of the semolina and on process parameters [3,4]. High-quality dry couscous grains are amber in color, uniform in size and lack a particular odor. They must have a high capacity to absorb water. After rehydration, couscous grains must be easy to remove with a fork, not sticky and not bulky. When chewed, they should remain cohesive, distinct and firm, with good taste and neutral flavor.

### 6.1. Biochemical Composition

Similar methods are used to measure the composition of dried couscous grains and native semolina. As couscous is made exclusively from semolina, the content of proteins, starch, lipids and fibers of dry couscous grains depends on the composition of the native semolina (Table 1) [3,4]. The water content of dry couscous depends on drying conditions. Thermal treatments during processing induce significant differences in the solubility of proteins and the extent of starch gelatinization. Although not a general rule, lower contents in gelatinized starch were found in some homemade types of couscous compared to one industrial type of couscous [36].

### 6.2. Size Distribution

**Figure 15:**
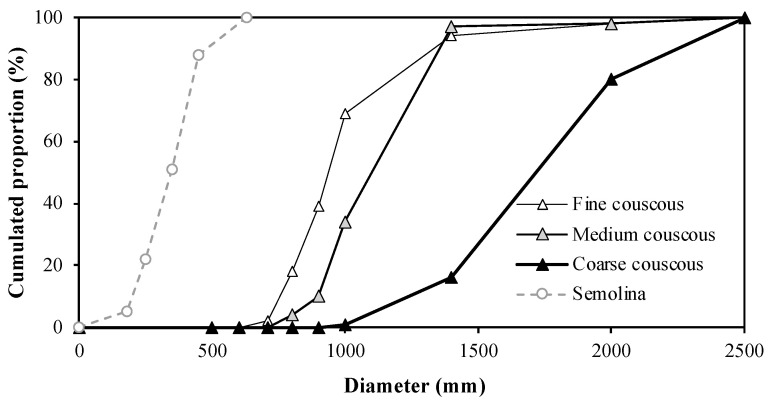
Typical examples of distribution curves of grain diameters for durum wheat semolina and different forms of couscous (adapted using data from Mezroua [50] and Lefkir [30], with permission from authors).

The diameter distribution of couscous grains is classically measured using a vertical vibrating sifter with sieves of decreasing mesh [3,4,5]. The *Codex Alimentarius* [1] specifies that the particle size distribution of dry couscous should be between 0.63 and 2 mm, with a tolerance of 6%. The particle size distribution of couscous follows a monomodal distribution (Figure 15). The relative position of the particle size distribution curves is specific to fine, medium or coarse couscous. Although not a general rule, some forms of artisanal couscous display finer grain size compared to medium industrial couscous, close to fine industrial couscous [12,39]. The diameter dispersion of couscous is relatively large and increases with the size (Figure 15). An increase in the extraction rate of semolina could cause a decrease in the median diameter of dry couscous [56]. At the mixing stage, an increase in the hydration level, a decrease in the water temperature or an increase in the duration of the mixing stage allows obtaining dry couscous with a greater diameter and lower dispersion [25,30,34,35]. The diameter distribution of the dried couscous mainly depends on the calibration stages after rolling and after drying.

### 6.3. Grain Shape and Microstructure

**Figure 16:**
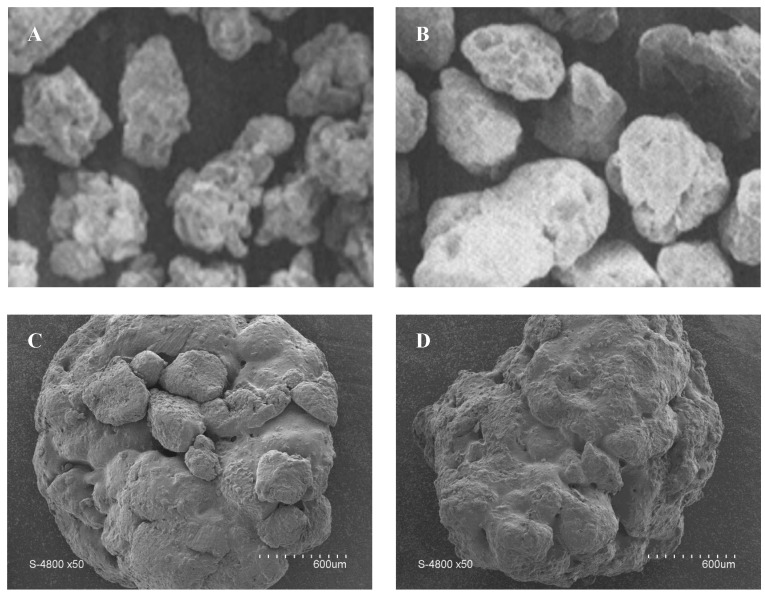
Scanning electron microscopy observation of the microstructure of homemade couscous (**A**) and commercial couscous (**B**) (adapted from Debbouz and Donnely [39]) and of couscous grains of industrial origin from rotary drum rolling (**C**) or plansichter rolling (**D**) (adapted from [4] with permission from authors).

The description of the shape of dry couscous grains is based on analysis of images obtained by optical or scanning electron microscopy [3,4,39,57]. A couscous grain appears as an approximately spherical granular object. The shape of grains was described using shape factors, such as circularity (0.68–0.73) and elongation (0.70–0.74) [25]. A couscous grain is formed by the assembly of durum wheat semolina particles that remain visible and partially melted to each other [54]. The melted bridges between particles contribute to the internal cohesion of grains. The quasispherical shape of couscous grains is the result of the mechanical stresses imposed during the process. Although not a general rule, grains of home-made couscous seem smoother and more uniform with rounded and oval shapes (Figure 16), unlike grains of industrial couscous which present more angular and heterogeneous shapes [12,26]. Dried couscous grains rolled in rotating drums seem more spherical than those rolled using plansichters.

### 6.4. Grain Porosity and Density

**Figure 17:**
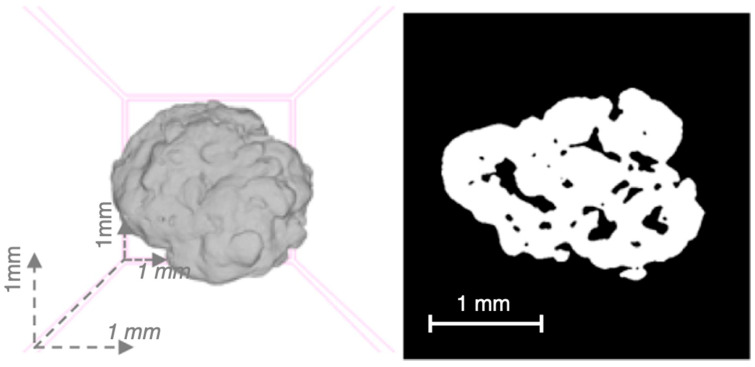
Typical examples of images calculated during the processing steps of XMT image analysis (adapted from [22], with permission from Elsevier, 2022).

The density of a dry couscous grain (1.39–1.41 g·cm^−3^) is slightly lower than the density of native semolina particles (1.46–1.48 g·cm^−3^) (unpublished data). The porosity of couscous grains has been determined by measuring the real density of the grains by X-ray microtomography (XMT) methods [22,58]. Couscous grains are slightly porous objects, with a compactness between 0.68 and 0.88. The porosity is due to the presence of entrapped air between the more or less melted semolina particles (Figure 17). The XMT closed porosity values (0.005–0.011) are 10 times lower than the internal porosity values (0.121–0.206) that were calculated from measured compactness values.

### 6.5. Bulk Density

The bulk density of couscous has been measured by filling a graduated cylinder [3,25,39]. Although not a general rule, values of bulk density range were found lower for some homemade types of couscous (0.60 g/cm^3^) than for one industrial type of couscous (0.79 g/cm^3^). The bulk density of couscous depends both on the compactness of the grains (true density) and on the air volume entrapped between the grains.

### 6.6. Color

The color of dry couscous grains can be quantified by instrumental methods using colorimeters and the color space parameters: L* (lightness), a* (red hue) and b* (yellow hue) [3]. Couscous grains are characterized by a high lightness L* (30–75), marked yellow hue b* (25–45) and low red hue a* (0–4) [25,26,33,39,47]. Although not a general rule, some artisanal types of couscous have been characterized by slightly higher yellow hue b* and lightness L* than one industrial type of couscous. The color of dry couscous grains greatly depends on the characteristics of the native semolina, in particular on the content of carotenoid and flavonoid pigments and on enzyme activities [3,5]. An increase in the extraction rate of the semolina leads to a decrease in the lightness of the dry couscous [58]. During processing, the hydration stage of the semolina initiates enzymatic reactions. Increasing the hydration level or the water temperature during mixing favors enzymatic reactions and reduces the yellow hue (b*) and lightness (L*) [25,30,35]. The degradation of the color can be slowed down by reducing the duration of the mixing stage and the number of recycles in the rolling stage [25,47]. The cooking stage also contributes to the degradation of carotenoid pigments, reduces the lightness (L*) and increases the yellow hue (b*) [25,47,59]. The drying stage conducted at high temperature (3 h at 95 °C) has a more marked effect on the color of the couscous compared to drying at low temperature (17 h at 55 °C) [25]. Granulometry of the dry couscous also greatly affects its color.

### 6.7. Hygienic Characteristics

According to the *Codex Alimentarius* [1], dried couscous must be free from microorganisms that may grow under normal storage conditions and must not contain any substance originating from microorganisms in quantities that may present a risk to health. When the homemade couscous is sun-dried, it can support the growth of *A. parasiticus* with production of aflatoxins during storage in the wet season [60,61]. Mold growth is possible if the product is not well dried or is poorly stored.

### 6.8. Rehydration Properties

Rehydration properties are important criteria of couscous grains. Determining rehydration properties of couscous grains is not an easy task, as many ways to rehydrate couscous exist. The methods used to determine rehydration properties of couscous grains are based on three complementary criteria: a swelling index which describes the water absorption capacity, a hydration time which describes the kinetics of water absorption and a solubility index which describes the loss of dry matter in water.

The swelling index is the relative increase in volume occupied by couscous grains immersed in an excess of water (at 25 or 100 °C), measured in a graduated tube, compared to the volume initially occupied at the time of immersion [3]. The test tube is placed in a water bath at a controlled temperature and the changes in couscous volume are recorded as a function of time [55]. Water absorption index can also be determined by introducing couscous and water in a centrifuge tube, shaking for 30 min and centrifuging at 2200 *g* for 10 min. The supernatant liquid is drained and the material remaining in the centrifuge tube is weighed to calculate the water absorption index. A great diversity in measured values of the swelling index for couscous exists, between 130% and 415% [35,39,49,62,63,64]. It is accepted that high swelling values are indicative of high-quality couscous [25,55]. Starch fraction plays a determinant role in the swelling index of the dry couscous [43]. The content of damaged starch of semolina has been supposed to impact the water absorption index [33,65]. The total lipid content of durum wheat semolina was correlated with the swelling index of couscous [49]. The protein content of the semolina has been partially negatively correlated (*r* = −0.571) with the swelling index of dry couscous [50]. A slight positive correlation was found between the swelling index and the bulk density of the couscous [47].

It remains difficult to discriminate between homemade couscous and industrial couscous by their swelling index values [39,47]. During mixing, an increase in the water temperature has negative effects on the dry couscous swelling [30,35]. The swelling index of couscous increases significantly with increasing hydration level during mixing, due to positive effects on the gelatinization mechanisms during the cooking stage [25]. The swelling capacity of couscous is proportional to the duration of the cooking stage [66]. A high correlation (*r* = 0.90) was found between the swelling index and the extent of starch gelatinization of couscous grains [67]. The drying temperature seems to have a negative effect on the swelling of couscous [25,68]. The insoluble glutenin content of dry couscous after the cooking and drying steps has been positively correlated (*r* = 0.73) with the swelling capacity [30].

The rate of water absorption by the dry couscous is often associated with the empirical term “optimal time” or “cooking time”, in comparison with the method of assessing the quality of pasta [3]. Rehydration time is assessed by measuring the time required for maximum water absorption by the couscous during the rehydration test [33,39]. A decrease in the particle diameter of native semolina results in a decrease in the rehydration time of dry couscous [33]. The time required for couscous to swell decreases with an increase in the water temperature during mixing [25,46]. Some homemade types of couscous were characterized by lower rehydration times due to the smaller particle size compared to one industrial type of couscous [39].

Rehydration of dried couscous in excess water can result in the solubilization of some dry matter in the water phase. The solubility index of couscous in water expresses the degree of disintegration of couscous [3]. The terms “cooking loss” and “degree of delitescence” are also used [33,49]. There is a great diversity in the published values of water solubility index for couscous, between 3% and 16% [39,49,63,69]. A low value of the water solubility index is generally associated with good-quality couscous. The water solubility index is correlated with the stickiness of the rehydrated couscous. The swelling index of couscous is inversely proportional to its water solubility index [47]. The use of fine semolina leads to couscous with high water solubility index [56,70]. The protein content of semolina is partly negatively correlated (*r* = −0.59) with the delitescence index of couscous [50]. The apolar lipid content is significantly correlated with the water solubility index [49]. It was difficult to discriminate between some homemade types of couscous and one industrial type of couscous by their water solubility index values [39,47]. During mixing, a decrease in the hydration level or in the water temperature reduces the water solubility index of the couscous [34,35]. The water solubility index of dry couscous has been correlated with the denaturation state of proteins after hydrothermal treatments. The content of insoluble glutenins in dry couscous was negatively correlated (*r* = −0.47) with the degree of delitescence [30,71].

### 6.9. Stickiness and Caking Index

The caking index is related to the aggregation of couscous grains after rehydration [3]. It can be determined by an instrumental method (proportion of grains with a diameter greater than 3 mm formed after rehydration and drying) or by sensory analysis. A low value of the caking index is an indicator of a high-quality couscous. There is a great diversity of values for the caking index (between 5% and 80%) in the literature [25,47,49,50,53,68,72]. The caking index is inversely proportional to the granulometry of the couscous: fine couscous is perceived as stickier than medium couscous. The caking index was positively correlated (*r* = 0.91) with the water solubility index. The solubilized dry matter at the time of rehydration contributes to the sticky character of the grains and favors their caking. The sticky character of couscous was associated with the extent of starch gelatinization and the possible diffusion of amylose chains on the surface of the couscous grains. An increase in cooking time increases the stickiness of couscous.

The use of fine semolina (instead of coarse semolina) results in dry couscous that is stickier and easier to disperse [33,56]. The stickiness of couscous decreases with increasing protein content and gluten index value of the semolina [33,70]. The extraction rate of the semolina has no significant effect on the caking index [56]. No correlation was found between the total lipid content of semolina and the caking index [49]. During mixing, increases in the water temperature, hydration level or mixing time result in increases in the stickiness and in the caking index values [25,30,34]. The drying temperature also affects the stickiness of the couscous [68,73]. Although not a general rule, some homemade types of couscous showed lower stickiness than one industrial type of couscous [39,47]. Belaïd et al. [46] showed that the incorporation of 1% monoglycerides during processing reduces the stickiness of couscous.

### 6.10. Texture Properties

Texture properties of couscous are described during its preparation and consumption by sensory analysis [3]. Texture qualities relate to the couscous ability to be fragmented with a fork and to its texture during chewing, with terms such as firmness, consistency, elasticity, smoothness, chewiness or stickiness [12,25,33,39,49,50,72]. Couscous of good culinary quality can be forked after rehydration; it should maintain its firmness it should have a not too firm consistency and a soft appearance and it should be easy to chew. An instrumental compression method was developed to evaluate the viscoelastic characteristics of a rehydrated couscous bed by transposing a method classically used for pasta [49,55,72]. However, the instrumental firmness is not correlated with sensory analysis [53]. The use of coarse (instead of fine) semolina results in firmer couscous grains [56]. Protein and total lipid contents of semolina were not correlated with the texture of the couscous [49]. Although not a general rule, a higher elasticity was found in some homemade types of couscous compared to one industrial type of couscous [26]. The incorporation of 1% monoglycerides during processing increases the firmness of couscous [46]. The firmness of the couscous is proportional to its granulometry: fine couscous is less firm than medium couscous.

### 6.11. Nutritional Characteristics

The nutritional qualities of rehydrated couscous are typical of a food based only on durum wheat semolina. Couscous is a source of proteins with “good” nutritional quality (except the low lysine content) and is a source of energy (350 kcal/100 g of dry matter) due to its high starch content. During chewing, couscous grains are not easily broken down and have slow rates of softening [74]. Couscous grains display a slightly higher absorption rate (glycemic index between 60 and 65) than pasta (glycemic index between 50 and 55). The porous granular structure, the high specific surface area and the lack of protein network around the starch granules in couscous grains are favorable for enzymatic attacks during digestion. The vitamin content of couscous is influenced by the thermal treatment. The content of vitamins (thiamin and riboflavin) was found to decrease with increasing steaming time [61]. As riboflavin is very sensitive to processing conditions, especially heat and light, its content in the traditional sun-dried samples tended to be lower than that in industrial samples.

## 7. Uses and Consumers of Couscous

In North Africa, couscous is an iconic food. It permits the expression of national identities and ways of life. It has religious and symbolic meanings. According to Habib Bouguiba, ex-leader of Tunisia, the border of Maghreb is marked by an imaginary line corresponding to a cultural boundary: to the east, the staple food is rice; to the west, the staple food is couscous [5,43]. Couscous is the dish that united the history and geography of Maghreb. The couscous dish allowed Morocco, Algeria, Tunisia and Mauritania to submit a joint file to UNESCO in order to obtain international recognition of the couscous dish as an intangible world heritage. Only the difficult political conditions that Libya is experiencing can explain Libya’s absence.

### 7.1. A Traditional Ethnic Food

In rural regions of Tunisia, women make the couscous alone at home or sometimes they ask their cousins or neighbors for help. Women choose a sunny day during summer and dedicate it to making a large quantity of couscous which covers the needs of their family throughout the year. This special day is named “Al Oula for one year”, which refers to joy and happiness [5]. Habitually, couscous-preparation knowledge was passed from mother to daughter and played a crucial role in North Africa’s patriarchal society. The know-how was an important “intangible” element of a young woman’s dowry.

Couscous is a staple in the Arab Maghreb region: it is the most popular in all the countries of the Maghreb. It symbolizes comfort, warmth and tradition. Women usually prepare couscous dish during a family celebration, and it is eaten during a family feast, thereby associating both the product and the dish with solidarity. Couscous accompanies the traditional Arabic weekend (Friday and Saturday); the end of Ramadan celebrations; and Muslim year, birth and wedding feasts. The association of couscous with these festivities also attaches it to the concepts of abundance, fertility, fidelity and Barakah (God’s blessing). While preparing couscous, women are used to making a kind of invocation and converse about religious facts, prosperity and positive feelings [5]. Couscous for interviewees with North African connections is first and foremost a dish that never needs to be paid for as it is a family dish and thus is not eaten in restaurants. A Mediterranean notion of sharing and valuing home and clan atmospheres is coupled with values that are centered not around money but around exchanges, and which see cultural prowess as having a sense of hospitality: to share a couscous is to be associated with other people and to express one’s attachment to the group [9]. The secret of couscous grains makes known the context of the Arab community in the host country, relating different aspects of the integration process, such as family relationships reasoned on solidarity-based and shared identity values [75]. The couscous, in the family, has a sociability function.

Couscous is well known to be consumed with a vegetable sauce. It can be prepared with vegetables, pulses and different types of meat, making the dish of couscous a complete one. When preparing the sauce, up to three vegetables can be included at the same time. The most commonly encountered are carrots, green beans, zucchini, potatoes, turnips, chard, cabbage, tomatoes, etc. Onion and garlic are added to the sauce as spices [6]. Couscous is a simple product that can also be prepared with a simple knob of butter or a little sugar and cinnamon.

### 7.2. Diversity of Couscous Market Offer and Consumption Patterns in the World

Ethnic consumption: In Algeria, there are more than 300 ways to prepare couscous, and spices and seasonings are one of the most important elements that distinguish the flavor of the dish from one country to another. There are as many recipes as there are villages, or even families in the Maghreb countries, each jealously guarding the secret of the recipe passed down from one generation to the other. Among “ethnic” consumers, couscous gives rhythm to daily and religious life. Couscous is the spiritual food of North Africans. In many North African families, the week cannot end without the Friday afternoon bowl of couscous after prayers. During Ramadan, mesfouf—couscous sweetened with cinnamon and raisins—is served before sunrise just before fasting, to keep everyone going until sunset. The end of Ramadan is celebrated in many homes by a more elaborate couscous than usual [76].

Couscous consumption: In France in 2020, the consumption of couscous was 1.5 kg per inhabitant [77]. Couscous is one of the favorite dishes of the French. In a 2006 survey, it came in second place after “blanquette de veau” and before “moules-frites” [78]. The traditional consumption can be apprehended with large packagings of 5 or 25 kg, purchased in France mainly by restaurant owners and North African consumers. A large part of the purchases in supermarkets of couscous in small packagings (0.5 or 1 kg) are made by consumers. In other European countries, the consumption of couscous is mainly made by the Maghrebian immigrant communities (Moroccan in Belgium and Germany, Tunisian in Italy). English and Polish markets are dominated by a so-called “modern” consumption of couscous (side dishes, tabbouleh, etc.).

From traditional dish to side dish: Today, couscous is available in traditional, canned, frozen and microwaveable forms. It has incorporated the codes of contemporary consumption: organic, fair trade, prepackaged meals, takeaway and so forth. It is served at canteens, restaurants, cafés, markets and catered events. It can even be the single unifying factor behind virtual communities, forums for couscous recipes and so forth. Couscous is a Mediterranean symbol of cultural interpenetration [9]. In the Western market, couscous is prepared due to its taste, quick preparation when presteamed and usage in salads (tabbouleh). In France, we observe a clear evolution in the consumption modes of couscous: 80% of the consumers used the grain in traditional dishes at the end of the 1980s; this proportion is only 60% today. Since the beginning of the 1990s, the consumption followed the rise of the tabboulehs in the delicatessen departments. Couscous grains are widely used in salads or as a vegetable side dish, such as rice or pasta. We have witnessed an important development of couscous called “flavored”, becoming widespread in the Anglo-Saxon countries, Great Britain and the United States [76]. Their success tends to prove that the traditional perception of the product is no longer the only one in the mind of the consumer.

International cultural cooperation and fusion: The “knowledge, know-how and practices related to the production and consumption of couscous” testify of a widely confirmed sociocultural importance in related countries. Throughout history, couscous has been able to travel and spread to other regions, such as Sahel and the Mediterranean islands. In the 20th century, it reached Europe, the Americas and Asia. It embodies and reflects successful cultural exchange and sharing. In France, the arrival of North African workers and repatriated French (after independence) in the mid-20th century largely contributed to popularizing the dish. It is in Sicily that since 1998 the “world championship of couscous”, the Couscous Fest, “festival of cultural integration”, takes place [79]. According to historians, *couscuz*, as it is called in Brazilian Portuguese, is a food that has its origins thousands of years ago among Berber peoples of North Africa, particularly in Morocco. It first crossed the Mediterranean to the Iberian Peninsula and then the Atlantic until it reached Latin America, where it was reinvented, rediscovered. Couscous in Brazil is a clear legacy of the Moroccan (Moorish) presence in Portugal. Today, couscous, which is celebrated every year on 19 March, World Couscous Day and the Feast of St. Joseph, is one of the main components of the intangible capital of the Northeast Region of Brazil [80]. In Brazil, couscous can be made with flour or starch from corn, rice or cassava. Salted and slightly moistened, the dough is marinated to incorporate the seasoning. It is steamed and can be enhanced with other ingredients, as is the custom in the Southeast, or simply accompanied by milk, eggs, butter or dried meat, as preferred in the Northeast.

### 7.3. Culinary Precisions as Explained by Science

*Is a couscoussier necessary?* The particularity of the preparation of couscous dish lies in the very particular kitchen utensil that is used: the “*couscoussier*”, a large metal pot in 2 parts: the steam basket above to cook the grains and the “big pot” at the bottom to cook the broth, meat and vegetables. This is the best way to cook the grains, as they are impregnated with the aromas of the broth. The steam will make them swell, making them light and more digestible.

*Can couscous be boiled?* Couscous is not cooked on the stove in boiling water. It can be steamed for a few minutes or prepared in a container simply by covering it with boiling water and seasoning with a drizzle of oil.

*How is light and easy-to-digest couscous made?* Couscous grains must be light and digestible. Couscous grains absorb the right amount of water necessary to swell, which will make them soft and not doughy. The grains can be coated with a thin film of fat provided by the oil with which we cover our hands to “roll” it.

## 8. Conclusions

Couscous, of Berber origin, has been eaten since at least the Middle Ages. If it is difficult to be definitive on its history, but everybody has fallen in agreement on this truth of couscous: “The best couscous, is the one of my mother”. Couscous cannot be summarized only by the emblematic dishes which contain it: couscous is much more than a dish; it is a moment, memories, traditions, know-how and gestures which are transmitted from generation to generation. There are as many couscous recipes as there are families and an infinite variety of nuances between regions, making couscous a true mirror dish of the societies where it is cooked. Outside the Maghreb region and outside Europe, most couscous is produced industrially: this may be responsible for the worldwide growth of couscous consumption. The complementary uses of couscous made by industrialists or at home fully meet the diversity of consumer needs, between tradition and innovation. Traditional couscous and industrial couscous are not in competition but ensure the perpetuity and expansion of consumption in the world.

## Figures and Tables

**Table 1:** Order of magnitude of protein and starch contents (g/100 g dry matter) of durum wheat semolina, coproducts generated during the manufacturing process and dry couscous grains (adapted from [3] with permission from Elsevier, 2022, adapted from [4] with permission from authors).

Composition	Durum Wheat Semolina	Wet Recyclates	Cooked and Dry Recyclates	Dried Couscous Grains
Water content	13–15	30–35	8–11	10–13
Gelatinized starch content	84–88	84–88	84–88	84–88
Gelatinized starch content	4–6	15–25	80–90	80–90
Total protein content	11–15	11–15	11–15	11–15
Soluble protein content	11–13	9–11	2–4	2–4
Total pentosan content	1–2	1–2	1–2	1–2
Soluble pentosan content	0–0.1	0–0.1	0–0.1	0–0.1
